# In this month’s Bulletin

**DOI:** 10.2471/BLT.26.001026

**Published:** 2026-10-01

In the editorial section, Nicola Abrescia et al. (658) list the research and development gaps for Bundibugyo virus disease. Oluwaseyi Muyiwa Egbewande et al. (659) discuss the global plastics treaty negotiations and their implications for health-care system use of plastic in the WHO African Region. 

## Bahrain, Kuwait, Oman, Qatar, Saudi Arabia, United Arab Emirates

### Health policy modelling 

Abdullah Alibrahim and Fadi El-Jardali (682–689) make a case for national capacity.

## Ethiopia

### Pharmaceutical manufacturing capacity

Kedir Tahir Hagos et al. (705–710) track a decade of improvements.

## Germany 

### A 10-minute bout requirement 

Julian Brummer et al. (673–681) compare physical activity estimates.

## India

### Diagnosis of enteric fever.

Kamini Walia et al. (714–716) argue for the elimination of Widal tests.

## Kenya 

### Malaria prevention and drug resistance 

Beatrice Rotich et al. (662–672) study seasonal chemoprevention.

## Global

### Population-based measures of oral health 

Nicole Rendell et al. (690–697) introduce a new module in risk factor surveys.

### Large language models in health care

Carlos Fernando Mourão and Luiz Eduardo Juliasse (698–704) frame requirements for accountability.

### Climate health research

Verina Wild et al. (711–713) explore issues of epistemic injustice.

